# Formulation and Evaluation of a Nutritionally Enriched Plant Protein-Based Matrix Using Low Temperature Extrusion Cooking—Air Drying Technology

**DOI:** 10.3390/foods14111846

**Published:** 2025-05-22

**Authors:** Ghaidaa Alharaty, Hosahalli S. Ramaswamy

**Affiliations:** Department of Food Science and Agricultural Chemistry, Macdonald Campus of McGill University, 21111 Lakeshore Road, Ste Anne de Bellevue, QC H9X 3V9, Canada; ghaidaa.alharaty@mail.mcgill.ca

**Keywords:** extrusion, antioxidants, protein, pea, rice, snack, healthy

## Abstract

Extrusion cooking is broadly used in the food industry due to its easiness and simplicity. In this study a twin-screw extruder is applied at 150 rpm and 24–28 °C for the production of a nutritionally enriched extruded matrix, where hypo-allergenic rice protein (RP) and pea protein (PP) were used in the entrapment of natural antioxidant blueberry powder. The higher-moisture-content extrusion (40% MC) used with protein mixture (75 PP:25 RP) reduced the output temperature from 61.97 °C to 55 °C, the expansion ratio from 1.26 to 1, and the rehydration ratio from 78.70% to 31.90%, when compared with low-moisture-content extrusion used with RP samples (25% MC). Combining RP and PP showed also an enhancement in the textural properties of the extruded samples where firmness and toughness increased to 1503 (g) and 1822 (g.s), respectively, and preserved the anthocyanin content and antioxidant activity during extrusion processing and subsequent finish air drying. Moreover, the addition of maltodextrin in low concentration (5%) enhanced the antioxidant activity and anthocyanin retention (by 98.59% in mixture samples after extrusion and 92.13% after drying) and improved the appearance and sensory properties of the extruded matrices including firmness, toughness, and the color of the added blueberry powder.

## 1. Introduction

In the recent years, the consumer demand for enriched and nutritious food products has increased due to advanced nutritional education and awareness [1]. This has resulted in a higher focus on the production of healthy snacks with added functional ingredients, using simple and low-cost technologies like extrusion cooking and air drying technology [2,3,4,5,6].

Extrusion cooking is generally defined as a high temperature short time (HTST) shear process, resulting in the application of high mechanical energy on raw materials in a relatively short period of time [1]. Extrusion is mainly used in the transformation of raw ingredients into modified intermediate and finished products [7]. The resulting high pressure, high temperature, and high shear prevailing inside the extruder can potentially eliminate biological hazards [8], and result in products of varying/desirable sensory properties. Generally, the extrudates need to be subjected to a finish drying process when they exit at a high water activity level due to incomplete removal of moisture [7,9], as in the case of a high-moisture and lower-temperature extrusion process. In order to result in a water activity level that yields shelf stability, these products either need to be air dried or can be preserved using freezing. In fact, this processing technology is broadly used, and shows multiple advantages in the food industry including process simplicity, high production yields, economic feasibility, reduced pollution, and oil-free cooking properties, as well as excellent opportunities for preparing a wide range of snack foods [3].

A screw-type extruder is made of three different zones that can be heated individually and independently, involving a feeding zone, compression zone, and cooking zone. Rotating screws transport the raw material or feed from the inlet to the final discharge through to the exit through a die placed at the end of the extruder. The friction between the feed and screw surface causes extrusion shearing (generally increasing with pitch reducing along the length) and heating of the conveyed material, resulting in a rapid increase in the temperature and pressure within the cooking zone, which reach their maximum before the product is forced out through the die [10]. The pressure difference often causes a significant amount of moisture to be removed from the extrudate and can even result in a dry expanded product when lower feed moisture contents and higher extrusion temperatures are used. At lower extrusion temperature and higher moisture levels, however, a finish drying step becomes necessary to lower the moisture content/water activity to a safe level for achieving safety and shelf stability.

Extruded products including pastas, cereals, and snacks are highly popular, while they are often labeled as unhealthy, calorie-dense produces, mainly for being made out of carbohydrates, sugars, and fats, leading to child as well as adult obesity. For the development of a new generation of healthy extruded food matrices, a primary focus is currently on replacing or reducing the carbohydrate and fat-dense fraction with legume proteins, antioxidants, and fibers. The extrusion of various legumes has recently been reported, including black beans [11], lentils [12], and pea proteins [13]. 

Rice protein (RP) is economically abundant and highly hydrophobic, with reduced allergenicity and high nutritional quality that can be compared to egg and milk proteins [14]. This vegan protein is mainly composed of hydrophobic glutelin—almost 80% of its composition [15]. The reduced solubility of RP in aqueous solutions has some negative influence on its use in the food formulation industry [14]. Furthermore, pea protein (PP) is a globular protein, with high digestibility and made out of a high proportion of globulins (up to 80%) with a small portion of albumin. Pea protein is also a hypo-allergenic vegan protein, with a high nutritional value and amino acid profile. However, this protein has low functionality including emulsification and gelling properties [15]. Studies suggest that combining pea protein and rice protein together can create a more complete protein profile and enhance the functional properties for the application/utilization of the proteins [16].

Furthermore, natural pigments and bio-active compounds including anthocyanins are currently used as healthy components in the production of new functional foods [3,17]. Anthocyanins (AAs) are water soluble polyphenols found in different fruits, vegetables, tubers, and flowers and they are responsible for their blue, red, and purple colors [1,18]. The use of these anthocyanin pigments is highly appealing to the consumer due to their potential health benefits, including their antioxidant and anti-inflammatory properties which help to enhance the protective effects against cancer and cardiovascular and neurological diseases. These pigments can also be used as natural color alternatives to synthetic dyes. Moreover, the incorporation of anthocyanins into the extruded matrices by simply adding fruit powders is an effective way to enhance the nutritional value and consumer acceptance of the extruded products [1]. However, the stability of anthocyanins is affected by multiple factors including oxygen, heat, and pH, which makes it challenging to incorporate them into many processed food matrices. Their lower thermal liability during processing and lower stability during storage pose a challenge for their use on a commercial scale [1]. It is hypothesized that anthocyanin entrapment within a plant protein-based matrix using extrusion technology will provide a protective effect to the added bio-actives and result in high antioxidant activity. To our knowledge there has been no study carried out up to now on the use of pea protein (PP) and rice protein (RP) combinations in the entrapment of berry powders using low-temperature high-moisture extrusion technology.

To further improve the encapsulation functions of used proteins, maltodextrin (MD), a carbohydrate polymer, has been studied for AA entrapment [19]. MD is generally used in the food industry as a filling and thickening agent. It is also used as an encapsulation agent, due to its ability to increase the stability of the encapsulated food ingredients, and it protects the active ingredients from oxidative damages. MD with elevated dextrose equivalency can also have higher encapsulation efficiencies since it is less leaky to oxygen [20]. Shinde and Ramaswamy (2021) used maltodextrin extensively to reduce the intake of solutes during microwave osmotic dehydration applications [21]. In other studies, MD has also been used for reducing oil intake by fried products during deep fat frying [22]. Studies have also showed that when combined with protein powders, MD can positively affect the matrix texturization and thermal stability of the extruded matrices due to the formed saccharide–protein interaction [23]. Moreover, MD has an impact on the cooking properties of the extruded matrices, which can enhance the textural properties of the extruded samples [24].

The objective of this study was, therefore, to evaluate and optimize the low-temperature high-moisture extrusion process to protect thermo-labile components and to enhance extrusion parameters and material properties to produce hypo-allergenic plant-based-protein-extruded matrices, with high nutritional value, antioxidant, and sensory properties.

## 2. Material and Methods

### 2.1. Materials

Rice protein and pea protein were obtained from Canadian Protein Co. Inc. (Windsor, ON, Canada). Maltodextrin of dextrose equivalent 18 (MD 18) was obtained from Sigma Aldrich Chemical Co. (Montreal, QC, Canada) and freeze-dried organic blueberry powder as a source of anthocyanin and natural colorant was purchased from LOOV organic (Loov Organic Store, Casper, WY, USA).

### 2.2. Experimental Design

Experimental design was developed using a central composite rotatable design software (CCRD), taking into consideration two main extrusion variables: the feed moisture content and the screw speed of the extruder [25], while barrel temperatures were set near room environmental conditions (24–28 °C) to reduce the degradation of added bio-actives. Several preliminary test runs were carried out using rice protein (RP) only, pea protein (PP) only, and protein mixtures (Mix). At a screw speed setting of 40 (200 rpm), the extruded samples were dry; at a screw setting of speed 20 (100 rpm), an unstable texturization was observed; while good protein texturization was observed at a screw speed setting of 30 (150 rpm).

### 2.3. Feed Preparation

Based on preliminary experiments, a feed moisture content of 25% was used with rice protein, 45% with pea protein, and 40% when a mixture of proteins (75:25 as PP:RP ratio) was used. Extrusion of rice protein was categorized as low-moisture-extrusion cooking (LMEC) [10–40% (*w*/*w*) water], while extrusion of pea protein and protein mixture were considered as high-moisture extrusion cooking [HMEC; 40–70% (*w*/*w*) water] [26]. Blueberry powder was added at 10% of the dry feed and mixed for 20 min using a Hobart Planetary Flour Mixer (Hobart Food Equipment Group, North York, ON, Canada) operating at a medium speed, before transferring the feed into the extruder. Effect of maltodextrin on the quality of extruded matrices was also evaluated. When used, maltodextrin (MD:18) was added at three different concentrations (5%, 10%, and 15%, *w*/*w*).

### 2.4. Extrusion Cooking and Air Drying

Extrusion cooking was performed using a co-rotating extruder (Double Screw Testing Extruder, DS32-II, Jinan Saixin Food Machinery, Shandong, China). In all three zones of the extruder (feeding zone, mixing or compression zone, and metering zone) the temperatures were set at 24–28 °C to prevent the degradation of added bio-actives. A constant screw speed of 150 revolutions per minute (rpm) (setting level 30) was used based on the preliminary test runs. The extruder was fed manually, keeping the flights of the screw fully covered and avoiding accumulation of the material in the hopper [25].

### 2.5. Sample Drying and Storage

**Figure 1:**
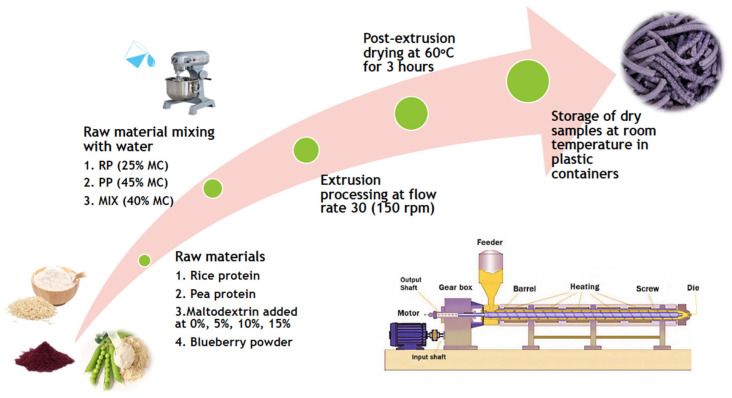
Methodology schematic used in the production of plant-based protein extrudates.

Following extrusion cooking, the extruded samples were collected and dried for 3 h using a cross-flow hot air dryer functioning at 60 °C to reduce moisture content and reach a lower moisture content to prevent mold growth during storage. Dry samples were stored in airtight plastic containers at room temperature and used for analysis [25]. Methodology used is schematically shown in Figure 1.

## 3. Testing

### 3.1. Output Temperature

Output temperature was measured using a non-contact infrared thermometer (−50~610 °C) for surface temperature measuring (Etekcity, CA, USA) to study the effect of speed change and moisture content on the output temperature of the extruded samples.

### 3.2. Expansion Ratio

Expansion ratio (ER) is defined as the ratio of the diameter of the extrudate to the diameter of the die. In order to determine the ER, 20 randomly selected cylindrical segments of each sample lot were used and the diameter was measured using a Vernier caliper and the mean value was used as compared to the die diameter [25].

### 3.3. Exit Moisture Content (%)

Moisture content was determined using a dry oven method. Samples were placed in triplicates in the drier oven at 120 °C for 8 h. Weight was recorded using an analytical balance before and after placing the samples in the oven [6,27] and the wet basis moisture content was calculated using Equation (1).

(1) $$Moisture\;content\;(\%)=\frac{\left(weight\;wet-weight\;dry\right)}{weight\;wet}\times \;100.$$

### 3.4. Water Holding Capacity (WHC)

Water holding capacity of proteins (the amount of water that can be absorbed by 1 g of protein) was determined using the methodology of Stone et al. (2015) [28], by adding 0.5 g of protein into 5.0 g of water in a 50 mL screw cap centrifuge tube. Samples were vortexed for 10 s every 5 min for a total of 30 min and then centrifuged at 1000× *g* for 15 min. The supernatant was poured out slowly and the remaining mass was weighed.

Water holding capacity was calculated by dividing the weight of sediment (weight gained by protein) by the original dry sample weight (g/g) of the sample [28,29]. 

### 3.5. Rehydration Ratio (RR)

Rehydration ratio measures the amount of liquid absorbed by dry extrudate. Each extruded sample after air drying (20 g) was weighed (M1) and added to 500 mL of water at 30 °C for 15 min. The water was drained and the rehydrated sample was weighed (M2) again [25]. The rehydration ratio (RR%) was obtained as shown in Equation (2).

(2) $$RR=\frac{\left(M2-M1\right)}{M1}\;\times \;100.$$

### 3.6. Texture Analysis

Texture analysis was performed using TA XT plus Texture Analyzer (Texture Technologies Corporation, Hamilton, MA, USA/Stable Micro Systems, Godalming, Surrey, UK). The methodology used was developed in our lab and has been used in several studies [7,12,21,25]. Seven to ten samples were selected from each lot, cut into equal pieces of ~5 cm length and 1.5 g of weight. A blade probe was used to measure firmness and toughness with return distance of 20 mm, return speed of 20 mm/s. The force displacement diagram was used to calculate the firmness and toughness texture parameters. Sample firmness signifies the maximum force recorded during first compression, while sample toughness determines the resistance against fracture [30,31]. 

### 3.7. Color Analysis and Appearance

The color characteristics of extruded samples before and after air drying were measured using a calorimeter (Tristimulus Minolta Chroma Meter) to determine the L* value (lightness), a* (green-red chromaticity) and b* (yellow-blue chromaticity), C value, H value, and ∆E. The C value, chroma, describes the purity or saturation of the color expressed in Equation (3). H value is the hue angle expressed in Equation (4) [32], with reference values for H° magenta red at 0/360°, yellow at 90°, bluish green at 180°, and blue at 270° [33]. Color differences between extruded samples and their respective raw formulations were expressed as ∆E. ∆E was expressed as shown in Equation (5) [6]. The calorimeter was calibrated using a white standard and readings were carried out at room temperature on powdered samples [1].

(3) $$\mathrm{Chroma}={{(a}^{2}+b^{2})}^{½}.$$

(4) $$\mathrm{Hue}\;\mathrm{angle}={tan}^{-1}(b/a).$$

(5) $$∆E\;=\;\surd ({\left(∆L\right)}^{2}+{\left(∆a\right)}^{2}+{\left(∆b\right)}^{2}).$$

### 3.8. Chemical Properties

#### 3.8.1. Anthocyanin Content

After phenolic compound extraction [6], anthocyanin content was determined using the pH-differential method. Briefly, 5 g of the sample was placed in aqueous acidified ethanol (50/50 water/acidified ethanol, *v*/*v*). Slurry was stirred for 2 min at room temperature and the final volume was brought to 100 mL before storage at 4 °C for 90 min to equilibrate. Slurry was centrifuged and filtered using Whatman no.1 filter papers. Potassium chloride buffer solution (0.025 M, pH 1.0) was prepared by dissolving 1.86 g of potassium chloride into 980 mL distilled water and pH was adjusted to 1.0 with HCl and then distilled water was added to make the volume 1 L. For the preparation of sodium acetate buffer solution, 54.43 of sodium acetate was dissolved in 980 mL of distilled water. pH was adjusted to 4.5 by adding HCl and distilled water was added to make up the volume to 1000 mL [34]. After extract solution preparation, 1 mL was transferred into 50 mL Falcon tubes, one for pH 1 and another one for pH 4.5 in duplicates. Samples were diluted to 50 mL with the respective buffers (pH 1 and pH 4.5). Absorbance was measured using a spectrophotometer (UV3100) at 510 nm and 700 nm. Samples were equilibrated in a dark environment at room temperature for 30 min [35,36]. Absorbance was measured in triplicates for each sample. The absorbance (A) and concentration of anthocyanin (mg/g) were calculated using Equations (6) and (7), respectively:

A= (A510 nm pH 1.0 − A700 nm pH 1.0) − (A510 nm pH 4.5 − A700 nm pH 4.5).(6)

(7) $$\mathrm{Anthocyanin}\;\mathrm{concentration}\;(\frac{mg}{g})=\;(\frac{\left(A\times MW\times DF\right)}{\left(\varepsilon \times L1\right)}.\frac{\left(mLex\right)}{\left(gs\right)}).$$

where A is absorbance, MW is molecular weight (449.2 g/mol), DF is dilution factor, ε is extinction coefficient of 26,900 M^−1^ cm^−1^, L1 is the cuvette pathway length [cm], mL ex is the volume of the original extract expressed in [mL], and gs is the sample mass (g) [37]. 

#### 3.8.2. Anthocyanin Retention Rate

Anthocyanin retention rate (%) was expressed as in Equation (8), where A_t_ is the anthocyanin content at different times and A_0_ is the anthocyanin content before treatment [38].

(8) $$\mathrm{Anthocyanin}\;\mathrm{Retention}\;\mathrm{Rate}\;(\%)=\frac{\left(At\right)}{\left(A0\right)}\;\times \;100.$$

#### 3.8.3. Antioxidant Activity

After phenolic compound extraction [6], briefly, 10 g of sample was mixed with aqueous methanol (50/50, *v*/*v*) and stirred for 2 min at room temperature and the final volume was brought to 100 mL before storage at 4 °C for 90 min to equilibrate. Slurry was centrifuged and filtered using Whatman no.1 filter papers. The scavenging activity for DPPH radicals was determined using spectrophotometric analysis [39]. For the preparation of 0.1 mM DPPH solution, 3.94 mg of DPPH was dissolved in 100 mL of anhydrous ethanol and mixed thoroughly at room temperature. For sample preparation, a 1 mL sample of extract solution was added to 3 mL of 0.04 mg/mL DPPH solution. Tubes were covered with parafilm, well shaken, and incubated for 30 min at room temperature. Absorbance was measured at 517 nm after 30 min of storage in the dark at room temperature. Radical scavenging activity was calculated using Equation (9).

Radical scavenging activity (%) = (1−(*Ai*−*Aj*)/*A* c) × 100. (9)

*A_i_* is the absorbance of the DPPH solution mixed with the sample, *A_j_* is the absorbance of 3 mL ethanol mixed with the sample (as a blank), and *A* _c_ is the absorbance of DPPH solution with 1 mL ethanol instead of the sample (as control) [40]. 

### 3.9. Statistical Analysis

A statistical analysis system (Analysis ToolPak in Excel) was used to carry out the one-way ANOVA at 95% level of confidence and 5% level of significance. The significance level used was (*p* < 0.05) [41]. 

## 4. Results and Discussion

### 4.1. Output Temperature

Output temperature was monitored after extrusion to study the effect of screw speed and feed moisture content on the warming up of the extrudate through the extruder, since the output temperature has a high correlation with the sensory properties of the extruded samples. Even though no heat was applied on the extrusion barrels, the friction of the screws caused pressure buildup, and temperature inside the extruder increased, leading to moisture evaporation and an increase in the output temperature of the extruded matrices.

In this study, a higher output temperature was obtained with RP samples due to the lower moisture content used (25%) in comparison with PP and mixture samples, which had higher moisture contents of 45% and 40%, respectively. Moreover, the increase in extruder screw speed from 100 rpm to 150 rpm and 200 rpm caused a progressive increase in the output temperature of all extruded samples, while higher output temperature was linked to RP due to the lower feed moisture content (Table 1). Overall, the exit temperature was below 70 °C, while the PP and mixtures had somewhat similar but >10 °C lower exit temperature profiles due to similar moisture contents. Lower moisture content, as in the RP formulations, creates more dry friction between the material and the counter rotating screw surface, and hence generates more heat. Due to lower moisture content, the product also will have a lower heat capacity and therefore the net effect is a significantly higher temperature rise. At the employed screw speed of 150 rpm, the exit temperatures were lower and also the differences were narrower, potentially reducing the thermal damage to the product quality parameters.

### 4.2. Exit Moisture Content (%)

Exit moisture content was measured after extrusion as well as after air drying to study the effect of the final product moisture content on the sensory and functional properties of the extruded samples. After extrusion cooking, moisture content decreased to 16.4% in RP samples, while the initial moisture content was about 25%. The extrusion process with all the heat generated within the extruder resulted in a significant loss in moisture content, but the product was still too wet to be shelf stable (very high water activity, a_w_ > 0.80). Following drying, the final moisture content was reduced to about 5%. The rice protein extruded product was somewhat open in structure and moisture was easily evaporated by the hot air during the drying process. Addition of maltodextrin (MD) at different levels significantly increased the moisture retention in the sample (26–29%, almost all of its initial value) and after air drying equilibrated to 10–12%, again significantly higher than without MD.

Results with pea protein (PP) varied significantly from RP. Since these were extruded at a higher moisture content (nominally 45%), the extruded product at the exit had 44–45% moisture level, retaining most of what was added. Following air drying, they equilibrated to much higher level than RP alone (5% vs. 14%), possibly due to differences in the protein structure and water holding capacities. Unlike with RP, the added MD did not make much difference with PP because the moisture retention was already at the maximum level. Furthermore, there was not much difference between the PP and PP-MDs after extrusion (44–45%) and drying (14–15%), possibly due to similar porosity/water holding characteristics between PP and MD. In Mix samples which had both RP and PP, the combined influence of the individuals was evident. The RP pulled down the MC levels both after extrusion exit and final air drying, since originally a lower feed moisture content was used (40% with Mix vs. 45% with PP). Additionally, added MD at different levels lowered the MC in Mix samples after extrusion (40–37%) and final air drying (10–9%) (Table 2). Overall, the final moisture content was about the same in MD moderated samples of RP and PP, demonstrating the advantages of adding MD into the mixture.

Extrusion cooking usually results in a slight reduction of the moisture content due to the evaporation of water during the process; however, the uneven moisture content after extrusion is mainly related to the higher moisture content applied on PP and Mix samples before extrusion. A higher quantity of water is needed for the texturization of PP and Mix samples due to the hydrophilic nature of PP, while a lower amount of water is needed for texturization of the hydrophobic RP [15].

Izalin et al. (2021) also reported a higher moisture content in soy protein- and pea protein-based extrudates due to the uneven input of moisture with the raw material placed in the feeder [42].

The different behavior of MD with PP and RP can also be related to the different chemical structures and functional properties of the proteins. Furthermore, maltodextrin is a hydrophilic carbohydrate, meaning it attracts moisture when bonded with a hydrophobic protein, which was responsible for the higher moisture content in RP-MD samples, while less effect is observed in PP-MD samples due to the similarity in their hydrophilic behaviors [43]. Increased hygroscopicity and water binding were also reported in extruded soybean–wheat protein mixture with 2% MD due to the effect of MD in increasing water binding and absorption [24]. Additionally, adding maltodextrin to pea protein during the production of meat alternatives enhanced the moisture content and texture of the extruded matrices [44]. Different behavior was observed in Mix-MD samples, where %MC declined with higher MD levels (Table 2). This behavior could be related to the role of MD in enhancing the stability of dry samples during storage by reducing their hygroscopicity. MD absorbs water, which at some levels allow the formation of a moisture protective layer on the surface of the hygroscopic particles, resulting in increased glass transition temperatures and reduced moisture content [45].

### 4.3. Expansion Ration (ER)

The expansion ratio (ER) is measured directly after extrusion to study the extent of puffing of the extruded samples, since ER is linked to the feed moisture content and matrices texturization. A small but significant difference was observed between RP, PP, and Mix samples, with the highest ER values recorded with RP samples (Table 3). The different ER values are mostly related to the moisture content of the feed, since low moisture content is used with RP (25% MC), while a high moisture content is used with PP and Mix samples (45 and 40% MC, respectively). Studies have related the increased moisture content to a decrease in the expansion ratio, which could be significant in our research and highly related to matrices texturization and blueberry powder entrapment.

It was reported that increasing feed moisture content is related to the decrease in the ER caused by the reduced viscoelasticity of the melt, which increases melt flow and facilitates its passage through the barrels with lower expansion [9]. Similar findings have been reported in extruded potato–chickpea-based snacks [46], extruded barley flour–carrot pomace snacks [47], and in extruded soy protein isolates and corn flour blends. A lower expansion ratio was observed at higher protein and feed moisture contents [25]. The feed with lower moisture content similar to RP in this study requires a higher dragging force, causing an increase in the pressure at the die of the extruder and a higher expansion [48]. Reduced feed moisture content can also show a higher viscosity which causes a greater pressure differential and an increase in the shear and residence time inside the extruder, resulting in a higher expansion of the extrudates [49]. Moreover, the decreased expansion ratio in PP and Mix samples could be caused by the insoluble fiber that might be available in PP in comparison with RP. Studies showed that fiber can minimize the ER by rupturing the cell walls and preventing the expansion of air bubbles within the extruded matrices [50,51]. 

### 4.4. Rehydration Ratio (RR)

Rehydration ratio (RR) measures the amount of liquid absorbed by dry extrudate. RR is an important parameter, since multiple extruded matrices are rehydrated prior to consumption including breakfast cereals and protein shake powders. A high rehydration ratio was observed in RP samples when compared to PP and Mix samples (78.70 vs. 39.00% and 31.90%, respectively) (Table 3). Studies have related the increased rehydration after drying to the lower initial moisture content of the feed, which leads to an increased ER and explains the increased rehydration in RP samples obviously resulting from the increased porosity of the product [25].

As expected, after adding maltodextrin, the RR significantly decreased in RP samples and reached the minimum value of 18% with the addition of 15% MD, while an increase in the RR was observed after adding MD to PP and Mix samples (Table 3). This different behavior could be first related to the decrease in the expansion ratio of the extruded RP samples after adding MD, since lower ER is attributed to result in a lower rehydration ratio [52]. Similar behavior was reported by Yu et al. (2013) [25]. The reduced moisture content in the feed of soy protein isolates and corn flour resulted in an increase in the rehydration rates of the dry extrudates, while an increase in the initial moisture content of the feed resulted in a lower rehydration [25]. In PP and Mix samples, however, the general trend was an increase in RR with an increased incorporation percentage of MD, reaching 88–90% values at the highest level of MD incorporation (15%). This is something desirable to have in an extrusion-dehydrated product, and the incorporation of MD increased the moisture absorption and retention during the rehydration process. This, however, did not arise from the effect of extrusion process the on the ER because the ER of all MD-added samples of both PP and Mix remained almost the same. This might have been influenced by the hydrophilic nature of MD which increased with an increase in MD concentration in the sample.

### 4.5. Water Holding Capacity (WHC)

Measuring WHC can help us understand the hydration properties of the extruded matrices, important parameters that can greatly affect the texture and appearance of the produce [53]. RP samples showed lower WHC values in comparison with the PP and Mix samples with the values 1.44 g/g vs. 2.71 g/g and 2.28 g/g, respectively. These may be somewhat consistent with their higher initial solids content, keeping the structure together, preventing moisture retention. After adding maltodextrin, the WHC of RP slightly increased and reached 1.90 g/g with 15% MD but was still significantly lower than with PP and Mix samples. RR values observed with PP and Mix samples were similar (Table 3). The difference in WHC between PP and RP samples probably resulted in the ability of pea protein to absorb water and is associated with its hydrophilic nature in addition to the added hydrophilic content of MD [29]. In comparison, the rice protein is composed of glutelin, while nearly 40% of its amino acid residues are more hydrophobic [14]. 

The increase in WHC of different RP, PP, and Mix samples after adding MD can again be due to the hydrophilic nature of MD. The MD molecules coat the protein molecules and form more water binding sites, which creates a larger hydrophilic surface and increases the hydrophilic behavior of the protein surface. Added MD can influence the bound water content of the extrudates [44]. Wheat gluten showed similar behavior to RP when combined with soy protein and pea protein. WHC of 1.4 g/g was recorded with wheat gluten due to its hydrophobic nature, while moderate WHC of 2.7 g/g was recorded with pea protein and high WHC of soy protein with a value of 4.2 g/g [29]. Higher WHC of soy protein isolates in comparison with pea protein isolate is also reported by Lee et al. (2023), since soy protein has more polar amino acids [53]. 

### 4.6. Textural Properties

**Figure 2:**
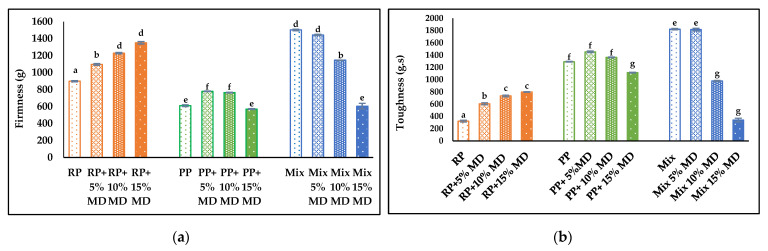
Texture analysis showing (**a**) firmness (g); (**b**) toughness (g.s) of RP, PP, and Mix samples after extrusion without MD and with 5%, 10%, and 15% MD. Different lowercase letters indicate a significant difference among samples (*p* < 0.05).

Firmness and toughness were evaluated as texture parameters and are influenced by the feed moisture content and extrusion process with RP, PP, and Mix with and without MD addition. Higher firmness was observed in RP and Mix samples in comparison with PP samples. After adding MD, a significant increase in the firmness of RP samples was observed, while small changes were observed with PP and a decrease in the firmness of Mix was observed (Figure 2a). In RP samples, the increased impact of heating may have been caused by the low moisture content of the feed, which could be the cause behind their increased firmness [26]. The poor WHC of the hydrophobic RP can also lead to moisture loss during extrusion and shows an impact on the texture of the extrudates [53]. It is not clear why the MD addition resulted in a consistent decrease in firmness in Mix samples—it appears the contributory effect of incorporated MD on RP firmness was pulled down by the contradictory effect of the addition of PP.

A different behavior was observed with measured toughness. The lowest toughness was observed in RP samples, which increased significantly several folds in PP and Mix samples. As with firmness, the toughness value of MD-incorporated RP increased, and Mix decreased, while the MD-PP samples showed lower values in comparison with Mix and a decreasing trend at higher MD levels (Figure 2b). Increased toughness in PP and Mix samples could also be related to the hydrophilic nature of the PP, which also increased when bonded with MD. High moisture extrusion used in the extrusion of PP and Mix samples (moisture content > 40%) resulted in extrudates with a highly elastic structure [43], which explains the increased toughness and rope-forming ability of PP and Mix samples when compared to RP samples produced using low moisture extrusion (moisture content < 40%). It was reported that water content of 55% has enhanced the flexibility or toughness of the extruded proteins [26]. 

Combining MD or other carbohydrates with protein powders during extrusions can improve the textural and structural properties of the extrudates [53]. However, at high MD concentrations, the increased water holding properties negatively affected the texturization and caused sample softening. Studies showed that the moisture content of the feed permits conformational changes on the extruded protein and shows a huge impact on the texture of the extruded matrices [43]. In Mix samples, an increase in the firmness and toughness was observed when MD was not incorporated. Similar behavior was reported by Khatkar et al. (2021) after adding soy protein (SP) to rice protein isolates (RPI) due to protein cross-linking and the change in the gelling ability of RPI and SP, which caused a change in textural parameters [54,55]. It was also reported that textural parameters were enhanced in soybean–wheat protein extruded mixtures with low MD concentration (2%), while texture deterioration was observed at higher MD levels due to a negative impact on the fiber structure formation [24]. 

### 4.7. Appearance and Color Analysis

#### 4.7.1. Appearance

**Figure 3:**
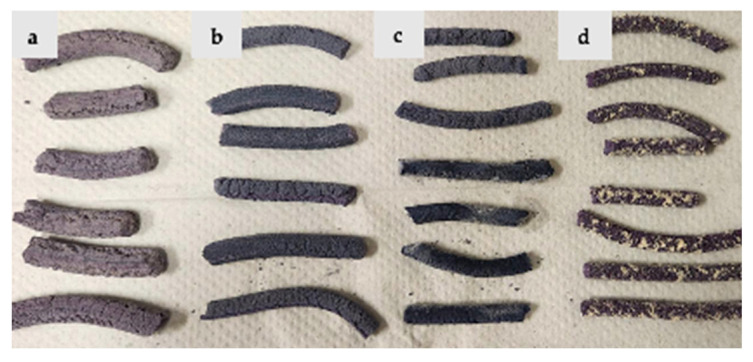
Appearance of (**a**) RP samples with no added MD; (**b**) RP samples with 5% MD; (**c**) RP samples with 10% MD; (**d**) RP samples with 15% MD.

**Figure 4:**
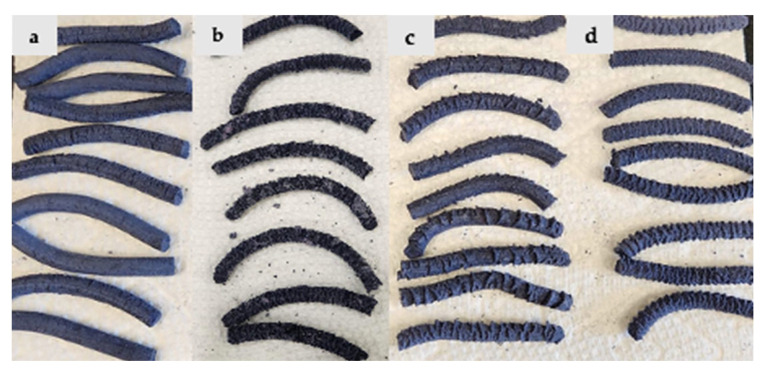
Appearance of (**a**) PP samples with no added MD; (**b**) PP samples with 5% MD; (**c**) PP samples with 10% MD; (**d**) PP samples with 15% MD.

**Figure 5:**
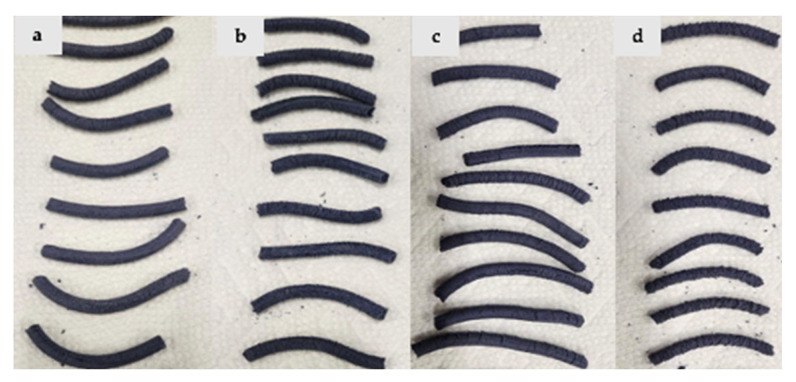
Appearance of (**a**) Mix samples with no added MD; (**b**) Mix samples with 5% MD; (**c**) Mix samples with 10% MD; (**d**) Mix samples with 15% MD.

The appearance of the extruded matrices is shown for rice protein (Figure 3), pea protein (Figure 4), and protein mixtures (Figure 5). Rice protein-based samples show a high expansion ratio and light purple-blue color formation (Figure 3). After adding MD, the expansion ratio was reduced, and the color of the samples was slightly enhanced. In the pea protein-based sample, the expansion ratio was lower, and the blue-purple color of the samples was stronger. However, at high maltodextrin concentration, texture deterioration was observed (Figure 4). In the mixture-protein sample, a low expansion ratio was observed with good texturization and a strong blue-purple color, while better texture and appearance was observed at a low MD concentration (5% vs. 10% and 15%) (Figure 5).

#### 4.7.2. Color Parameters

Color analysis was performed after both the initial extrusion cooking (wet samples) and the subsequent drying (dry samples) process to study the processing effects on the color parameters. Initially, L* value (brightness) decreased after adding the blueberry powder to RP, PP, and Mix samples due to the dark pigmentation on the normally whitish protein powders. After extrusion, much lower L* values were observed in PP samples in comparison with RP and Mix samples and this could be related to the primarily darker color of rice protein in comparison with pea protein (L* value of 50 in RP prior to extrusion vs. L* value of 22 in PP prior to extrusion) or related to the lower moisture content of the feed of RP extrudates [25]. The extrusion process itself could have also reduced the lightness of the samples (due to the thermal effects on pigments as well as surface proteins). Reduced lightness was also observed after drying (Table 4), which could be related to the Maillard reaction that happens during extrusion cooking or drying—both relatively high-temperature processes with relatively lower moisture content and active Maillard reaction components (sugar and amino acids) [56]. 

The a* values were positive for RP and Mix samples, which indicated that more red color was formed during the process, with RP showing the highest redness. I think the red color formation was maximum in RP and since Mix also has a good proportion of RP, it also had the presence of redness. In PP samples, the a* values were negative, indicating that the sample had a greenish color appearance instead of red. This meant that the red component was either destroyed or masked by the greenish appearance. After air drying, a* value components increased in all RP, PP, and Mix samples, showing an increase in the relative redness of the samples, resulting from the more favorable Maillard-reaction-forming conditions during the drying (dry and elevated temperatures) (Table 4).

For b*value, a positive value was observed in RP prior to extrusion, indicating the presence of a yellow component in the sample. After extrusion, the b* values turned negative in RP, PP, and Mix samples due to the disappearance of yellow and appearance of a deeper bluish color formation, with PP and Mix samples showing the highest values for blue color, possibly contributed more by PP. After air drying, Mix samples showed the more intense blue color formation with a decrease in b* values (Table 4).

C* value was higher in PP and Mix samples in comparison with RP samples, again indicative of a higher intensity blue color in PP and Mix samples. After drying, the color intensity increased further in RP and Mix samples, with higher values recorded with Mix samples (Table 5). A decrease in the color intensity in blueberry and grape pomace during extrusion cooking was reported [57]. 

The hue angles of RP, PP, and Mix samples revealed the blue-purple angle on the hue angle wheel (275–300°). Significant differences were observed between RP, PP, and Mix samples after extrusion and drying, with the Mix samples being more into the blue degree, while the PP and RP samples were more into the purple color degree (Table 5). Overall, in the extrusion-dried samples the retention of blue color of the added colorant was clearly retained, with better performance demonstrated by the Mix product groups.

**Figure 6:**
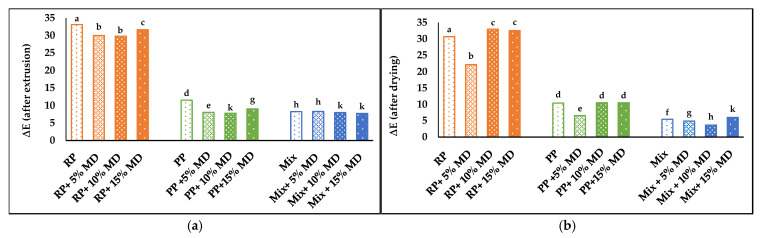
Color difference (∆E) (**a**) after extrusion; (**b**) after air drying of RP, PP, and Mix samples without MD and with 5, 10, and 15% MD. Different lowercase letters indicate a significant difference among samples (*p* < 0.05).

The total color difference (∆E) was also measured. After extrusion and drying, RP samples showed the highest color change, while PP and Mix samples showed better results. The lowest ∆E values were observed in Mix samples, which indicated the highest color retention (Figure 6a,b). Similar behavior was reported by Schmid et al. (2021) during extrusion processing of chokeberries [58], and during extrusion processing of Goji berry powders [56].

### 4.8. Chemical Components

#### 4.8.1. Anthocyanin Content (AA) and Anthocyanin Retention (%)

Anthocyanin content (AA) and radical scavenging activity (%) were measured after extrusion (wet samples) and after the subsequent drying (dry samples). Control was considered as the dry feed mixture powder (RP or PP or Mix) with added blueberry powder (BBP) before extrusion.

Some anthocyanins deterioration was expected during the extrusion process due to the effect of the high-shear heat reactions on the these thermally sensitive pigments [58]. A significant decrease in the anthocyanin content was observed in RP samples. When MD was added, anthocyanin retention significantly increased and reached 2.01 mg/g with 15% MD. In PP samples, a significant decrease in AA was also observed after extrusion and also with added MD. In Mix samples, AA decreased after extrusion to 2.75 mg/g, with a significant loss at higher MD concentrations (Table 6).

**Figure 7:**
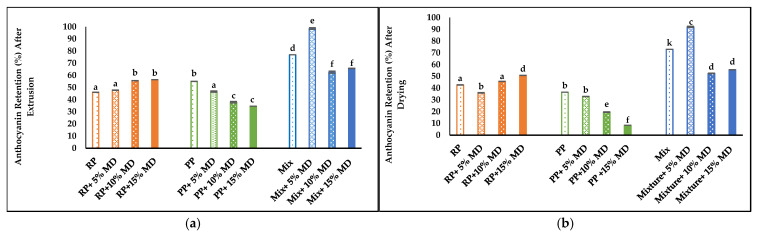
Anthocyanin retention (%): (**a**) after extrusion; (**b**) after air drying.

Anthocyanin retention after extrusion is shown in (Figure 7a), with the highest retention in Mix samples. Moreover, anthocyanin retention decreased in all RP, PP, and Mix samples after drying, with the highest retention also observed in Mix samples (Figure 7b). The increased moisture content used in PP and Mix samples caused an increase in anthocyanin retention in comparison with RP samples. Moreover, added MD enhanced the entrapment and retention of anthocyanin. However, in PP samples, low anthocyanin retention was observed after extrusion and drying due to texture deterioration (Figure 7a,b).

Hirth et al. (2014) also reported a decrease in the anthocyanin content in starch-based foods after extrusion cooking, with anthocyanin retention of 50% at reduced barrel temperature and at higher feed moisture content due to the decreased viscosity caused by the higher barrel temperatures [1]. High destruction of anthocyanin (90%) in cereals with blueberry powders was reported [59]. Blueberry and grape anthocyanin deterioration using extrusion cooking by 90% and 74%, respectively, was also reported [57]. The increase in moisture content can also decrease the mechanical energy and reduce anthocyanin deterioration [60]. 

#### 4.8.2. Radical Scavenging Activity

A decrease in the radical scavenging antioxidant activity was observed in all samples after extrusion cooking. The RP sample showed the highest deterioration in antioxidant activity, where minimal deterioration was observed in Mix samples when compared with the control. Moreover, higher antioxidant values were obtained with added maltodextrin. The higher antioxidant activity in Mix and PP samples could be related to the higher initial moisture content in the feed, which could have reduced the temperature rise during extrusion and minimized the deterioration of the antioxidant activity. At a higher feed moisture content, gentle processing can take place, resulting in higher anthocyanin retention. On the other hand, a lower moisture content in the feed results in higher shearing effects during extrusion, which causes the destruction of anthocyanin and antioxidant activities [61,62]. 

After drying, an increase in the antioxidant activity was observed in RP and Mix samples with added maltodextrin, which might be caused by Maillard reaction occurrence during sample drying [63]. Similar results were reported by Camire et al. (2007), where an increase in the antioxidant activity with low anthocyanin content was reported. The increase in antioxidant properties could be caused by the Maillard browning, caused by extrusion heat and friction in cereals with added fruit [59].

Antioxidant activity in PP samples is reduced after drying at high MD concentrations, which might be caused by the decrease in the anthocyanin content, reduced firmness, and texture deterioration of PP samples, which facilitated the decline in antioxidant activity (Table 6). Increased antioxidant activity of extruded goji berries after extrusion was also reported [6]. This behavior could be related to the liberation of phenolic compounds from their cell wall linkage cites or to the antioxidant activity of the produced Maillard products [56]. It is reported that hydrolyzed starches such as maltodextrin increase the microencapsulation efficiency of bio-actives due to their high solubility [64], which explains the increase antioxidant activity observed in RP samples with 5%, 10%, and 15% MD and in Mix samples with 5% MD. However, different behavior is observed in PP samples with added MD, and it could be caused by the texture deterioration of PP samples at high MD concentrations.

## 5. Conclusions

The effect of extrusion cooking processing parameters on the sensory and chemical properties of extruded plant-protein-based matrices were evaluated. High-moisture-content extrusion used with pea protein and Mix samples reduced the output temperature, expansion ratio, and rehydration ratio of the extruded matrices when compared with low-moisture-content extrusion used with rice protein. Moreover, the high moisture of the feed helped preserve the anthocyanin content and antioxidant activity of PP and Mix samples after extrusion processing and drying, with the highest anthocyanin retention observed in Mix samples. Furthermore, added maltodextrin enhanced the antioxidant activity, anthocyanin retention, and sensory properties of the extruded protein Mixture, while a negative influence on pea protein-based samples was observed due to texture deterioration and a decrease in firmness at high maltodextrin concentrations. In this study, a successful entrapment of blueberry powder within a hypo-allergenic extruded mixture (made of rice protein and pea protein with a low concentration of maltodextrin) is reported.

The study provides conceptual opportunities for developing high-value, nutrient-dense snack foods by mixing plant-based proteins from different sources and employing high-moisture, low-temperature extrusion followed by finish air drying under mild conditions. The nutritional profile can be custom designed to suit the target needs.

## Figures and Tables

**Table 1:** Effect of moisture content and extruder screw speed on the output temperature of extruded samples.

Sample	Moisture Content (%)	Output Temperature (°C) at 100 rpm	Output Temperature (°C) at 150 rpm	Output Temperature (°C) at 200 rpm
Rice protein (RP)	25	55.2 ± 1.28 ^aA^	62.0 ± 1.40 ^bD^	72.0 ± 1.20 ^cF^
Pea protein (PP)	45	48.9 ± 1.12 ^aB^	50.9 ± 0.93 ^aE^	60.9 ± 1.10 ^bK^
Mixture (Mix)	40	46.2 ± 2.33 ^aC^	55.0 ± 0.61 ^bE^	61.2 ± 0.87 ^bK^

Different lowercase letters within a row indicate a significant difference among samples with similar compositions due to screw speed change (*p* < 0.05). Different uppercase letters within a column indicate a significant difference among samples at similar screw speed due to composition change (*p* < 0.05).

**Table 2:** Moisture content of (%MC) of RP, PP, and Mix samples with added MD after extrusion (wet samples) and after drying (dry samples).

Sample Name	MC (% Wet Basis)	
	Wet Samples	Dry Samples
RP-based samples		
RP	16.43 ± 0.82 ^a^	4.96 ± 0.52 ^a^
RP + 5% MD	29.13 ± 1.92 ^b^	11.88 ± 1.12 ^b^
RP + 10% MD	26.20 ± 4.84 ^c^	11.61 ± 0.94 ^b^
RP + 15% MD	28.53 ± 2.78 ^f^	10.81 ± 0.30 ^f^
PP-based samples		
PP	44.21 ± 0.21 ^d^	13.99 ± 0.05 ^c^
PP + 5% MD	44.36 ± 0.24 ^d^	14.31 ± 0.22 ^c^
PP + 10% MD	44.86 ± 0.16 ^d^	14.57 ± 0.24 ^c^
PP + 15% MD	44.08 ± 0.54 ^d^	14.81 ± 0.08 ^c^
Mixture-based samples		
Mix	40.75 ± 0.40 ^e^	10.01 ± 0.42 ^f^
Mix + 5% MD	39.42 ± 1.43 ^n^	9.84 ± 0.05 ^f^
Mix + 10% MD	38.16 ± 1.01 ^k^	9.54 ± 0.20 ^f^
Mix + 15% MD	36.81 ± 0.24 ^m^	9.15 ± 0.65 ^e^

Different lowercase letters within a column indicate a significant difference among samples with different compositions (*p* < 0.05).

**Table 3:** Expansion ratio (ER), rehydration ratio (RR), and water holding capacity (WHC) of RP, PP, and Mix samples with added MD after extrusion.

Sample Name	ER	RR (%)	WHC (g/g)
RP-based samples			
RP	1.26 ± 0.60 ^a^	78.70 ± 1.21 ^a^	1.44 ± 0.40 ^a^
RP + 5% MD	1.20 ± 0.40 ^a^	49.00 ± 2.31 ^b^	1.61 ± 0.10 ^b^
RP + 10% MD	1.16 ± 0.04 ^b^	34.00 ± 2.12 ^c^	1.70 ± 0.04 ^c^
RP + 15% MD	1.00 ± 0.05 ^d^	18.00 ± 1.60 ^d^	1.90 ± 0.04 ^d^
PP-based samples			
PP	1.10 ± 0.05 ^c^	39.00 ± 2.11 ^f^	2.71 ± 0.08 ^f^
PP + 5% MD	1.10 ± 0.05 ^c^	59.00 ±1.73 ^m^	2.75 ± 0.20 ^f^
PP + 10% MD	1.10 ± 0.05 ^c^	86.50 ±1.85 ^k^	2.78 ± 0.03 ^g^
PP + 15% MD	1.10 ± 0.05 ^c^	91.20 ±2.20 ^n^	2.81 ± 0.04 ^g^
Mix-based samples			
Mix	1.00 ± 0.05 ^d^	31.90 ±1.14 ^c^	2.28 ± 0.11 ^k^
Mix + 5% MD	1.00 ± 0.05 ^d^	58.00 ±2.20 ^m^	2.26 ± 0.20 ^k^
Mix + 10% MD	1.00 ± 0.05 ^d^	86.00 ±1.75 ^k^	2.82 ± 0.10 ^m^
Mix + 15% MD	1.00 ± 0.05 ^d^	88.00 ±1.70 ^k^	2.85 ± 0.10 ^m^

Different lowercase letters within a column indicate a significant difference among samples with different compositions (*p* < 0.05).

**Table 4:** Color analysis (L*, a*, and b*) of RP, PP, and Mix samples without MD and with 5%, 10%, and 15% MD.

	L* Value		a* Value		b* Value	
Sample	Wet	Dry	Wet	Dry	Wet	Dry
RP before extrusion	50.04 ± 1.11 ^a^	NE	1.50 ± 0.10 ^a^	NE	9.45 ± 0.90 ^a^	NE
RP	24.50 ± 1.32 ^bA^	22.82 ± 0.80 ^aB^	1.98 ± 0.22 ^bC^	2.68 ± 0.14 ^bD^	−3.35 ± 0.31 ^bE^	−4.74 ± 0.09 ^bG^
RP + 5% MD	24.39 ± 1.10 ^bA^	21.97 ± 1.00 ^bB^	2.43 ± 0.72 ^cC^	2.32 ± 0.21 ^cD^	−3.07± 1.11 ^cE^	−4.8 ± 0.72 ^bG^
RP + 10% MD	23.40 ± 1.58 ^cA^	20.20 ± 1.31 ^cB^	2.50 ± 0.10 ^cC^	2.10 ± 0.20 ^dD^	−3.89 ± 0.19 ^dE^	−4.59 ± 0.55 ^cG^
RP + 15% MD	21.03 ± 0.61 ^dA^	20.45 ± 1.12 ^kB^	2.84 ± 0.06 ^dC^	2.3 ± 0.10 ^cD^	−3.14 ± 0.12 ^cE^	−4.10 ± 0.10 ^dG^
PP before extrusion	21.47 ± 1.13 ^d^	NE	−0.28 ± 0.03 ^e^	NE	−1.32 ± 0.21 ^e^	NE
PP	11.51 ± 0.50 ^fA^	11.12 ± 1.10 ^vA^	−0.47 ± 0.08 ^kC^	0.22 ± 0.04 ^eD^	−7.12 ± 0.13 ^fE^	−0.49 ± 0.05 ^eG^
PP + 5% MD	15.00 ± 1.11 ^gA^	11.20 ± 0.22 ^wB^	−0.62 ± 0.05 ^gC^	0.43 ± 0.6 ^fD^	−7.27 ± 0.20 ^gE^	−0.85 ± 1.12 ^fG^
PP + 10% MD	16.51 ± 1.11 ^hA^	11.01 ± 0.50 ^dB^	−0.62 ± 0.06 ^gC^	0.41± 0.04 ^fD^	−7.32 ± 0.29 ^hE^	−0.55 ± 0.41 ^kG^
PP + 15% MD	16.27 ± 1.30 ^hA^	11.00 ± 0.88 ^dB^	−0.60 ± 0.08 ^gC^	0.40 ± 0.10 ^fD^	−8.73 ± 0.40 ^kE^	−0.62 ± 0.40 ^mG^
Mix before extrusion	33.10 ± 1.10 ^k^	NE	0.80 ± 0.11 ^f^	NE	−6.10 ± 0.30 ^m^	NE
Mix	24.89 ± 0.59 ^wA^	28.09 ± 1.00 ^jB^	0.57 ± 0.65 ^hC^	1.24 ± 0.24 ^gD^	−5.39 ± 1.00 ^nE^	−8.27 ± 0.40 ^nG^
Mix + 5% MD	24.81 ± 1.00 ^wA^	28.36 ± 0.70 ^eB^	0.64 ± 1.10 ^vC^	0.58 ± 1.00 ^hD^	−5.78 ± 0.58 ^wE^	−7.40 ± 0.62 ^wG^
Mix + 10% MD	25.13 ± 0.22 ^nA^	29.61 ± 0.21 ^fB^	0.69 ± 0.43 ^vC^	0.72 ± 0.54 ^wD^	−6.16 ± 1.31 ^mE^	−7.38 ± 1.10 ^wG^
Mix + 15% MD	25.39 ± 1.00 ^kA^	27.87 ± 0.55 ^gB^	0.31 ± 1.22 ^wC^	1.27 ± 0.81 ^nD^	−5.84 ± 0.23 ^wE^	−8.91 ± 1.28 ^vG^

Different lowercase letters within a column indicate a significant difference among samples with different compositions (*p* < 0.05). Different uppercase letters within a row indicate a significant difference among samples due to air drying (*p* < 0.05). NE in the table signifies not evaluated.

**Table 5:** Color analysis (C and H value) of RP, PP, and Mix samples without MD and with 5%, 10%, and 15% MD.

	C*		H	
Sample	Wet	Dry	Wet	Dry
RP before extrusion	9.57 ± 0.18 ^a^	NE	80.68 ± 0.70 ^a^	NE
RP	3.89 ± 0.38 ^bA^	5.44 ± 0.10 ^bB^	300.53 ± 0.8 ^bC^	299.56 ± 0.79 ^bC^
RP + 5% MD	4.54 ± 0.72 ^cA^	5.95 ± 0.10 ^cB^	300.82 ± 0.2 ^bC^	282.84 ± 0.65 ^cD^
RP + 10% MD	4.62 ± 0.21 ^dA^	4.93 ± 0.55 ^dB^	302.69 ± 0.51 ^bC^	291.46 ± 1.53 ^eD^
RP + 15% MD	4.24 ± 0.08 ^vA^	4.70 ± 0.14 ^eB^	312.10 ± 1.46 ^eC^	299.29 ± 0.58 ^bD^
PP before extrusion	1.36 ± 0.20 ^f^	NE	257.95 ± 1.8 ^d^	NE
PP	7.13 ± 0.08 ^eA^	0.54 ± 0.12 ^fB^	266.22 ± 0.64 ^eC^	294.61 ± 2.32 ^dD^
PP + 5% MD	7.09 ± 0.40 ^eA^	0.77 ± 0.32 ^gB^	266.39 ± 1.23 ^eC^	284.29 ± 0.70 ^cD^
PP + 10% MD	7.35 ± 0.27 ^kA^	0.76 ± 0.32 ^gB^	265.15 ± 0.84 ^eC^	293.23 ± 0.70 ^fD^
PP + 15% MD	8.75 ± 0.44 ^gA^	1.10 ± 0.54 ^kB^	266.06 ± 0.69 ^eC^	294.07 ± 0.54 ^dD^
Mix before extrusion	5.50 ± 0.40 ^k^	NE	276.00 ± 0.55 ^d^	NE
Mix	5.42 ± 1.00 ^kA^	8.36 ± 1.10 ^wB^	276.02 ± 1.19 ^dC^	278.55 ± 0.55 ^wD^
Mix + 5% MD	5.81 ± 0.40 ^wA^	7.41 ± 1.10 ^vB^	276.38 ± 2.19 ^dC^	274.49 ± 0.98 ^kD^
Mix + 10% MD	6.20 ± 0.57 ^vA^	7.42 ± 0.81 ^vB^	276.42 ± 0.64 ^dC^	275.57 ± 1.11 ^kD^
Mix + 15% MD	5.85 ± 1.10 ^wA^	9.01 ± 0.81 ^mB^	273.03 ± 1.64 ^dC^	278.11 ± 0.98 ^wD^

Different lowercase letters within a column indicate a significant difference among samples with different compositions (*p* < 0.05). Different uppercase letters within a row indicate a significant difference among samples due to air drying (*p* < 0.05). NE in the table signifies not evaluated.

**Table 6:** Anthocyanin content (mg/g) and radical scavenging activity (%) of extruded samples after extrusion and air drying.

Sample	Anthocyanin Content (mg/g)		Radical Scavenging Activity (%)	
	Wet Sample	Dry Sample	Wet Sample	Dry Sample
Control	3.56 ± 0.03 ^a^	NE	73.54 ± 0.26 ^a^	NE
RP-based samples				
RP	1.64 ± 0.03 ^bA^	1.52 ± 0.07 ^bB^	64.33 ± 0.18 ^cA^	55.02 ± 0.11 ^aB^
RP + 5% MD	1.70 ± 0.06 ^fA^	1.29 ± 0.10 ^cB^	65.62 ± 0.90 ^dA^	65.69 ± 0.28 ^bA^
RP + 10% MD	1.98 ± 0.02 ^cA^	1.63 ± 0.03 ^dB^	66.90 ± 0.90 ^mA^	67.17 ± 0.22 ^cB^
RP + 15% MD	2.01 ± 0.01 ^cA^	1.81 ± 0.01 ^eB^	69.71 ± 0.18 ^bA^	72.30 ± 1.10 ^kB^
PP-based samples				
PP	1.96 ± 0.01 ^cA^	1.30 ± 0.04 ^cB^	73.54 ± 1.10 ^aA^	57.65 ± 0.69 ^dB^
PP + 5% MD	1.66 ± 0.1 ^bA^	1.17 ± 0.2 ^kB^	72.40 ± 0.11 ^wA^	55.14 ± 1.69 ^aB^
PP + 10% MD	1.35 ± 0.04 ^dA^	0.70 ± 0.02 ^fB^	65.23 ± 1.62 ^dA^	50.80 ± 1.82 ^mB^
PP + 15% MD	1.23 ± 0.07 ^kA^	0.30 ± 0.02 ^wB^	60.96 ± 0.23 ^kA^	49.47 ± 0.70 ^mB^
Mix-based samples				
Mix	2.74 ± 0.02 ^mA^	2.60 ± 0.01 ^kB^	70.10 ± 1.27 ^bA^	74.43 ± 1.29 ^gB^
Mix + 5% MD	3.51 ± 0.10 ^aA^	3.28 ± 0.38 ^mB^	72.70 ± 1.48 ^jA^	78.83 ± 0.90 ^vB^
Mix + 10% MD	2.23 ± 0.02 ^gA^	1.87 ± 0.19 ^eB^	73.51 ± 1.48 ^aA^	74.62 ± 0.22 ^gB^
Mix + 15% MD	2.34 ± 0.06 ^hA^	1.98 ± 0.19 ^jB^	73.75 ± 1.80 ^aA^	74.20 ± 1.17 ^gB^

Different lowercase letters within a column indicate a significant difference among samples due to composition changes between (*p* < 0.05). Different uppercase letters within a row indicate a significant difference among samples due to air drying (*p* < 0.05). NE in the table signifies not evaluated.

## Data Availability

The original contributions presented in the study are included in the article; further inquiries can be directed to the corresponding author.
